# Experimental and Density Functional Theory Simulation Research on PdO–SnO_2_ Nanosheet Ethanol Gas Sensors

**DOI:** 10.3390/s24154970

**Published:** 2024-07-31

**Authors:** Hao Wu, Jianwei Zhang, Huichao Zhu, Xiaogan Li, Hongxu Liu, Zhenan Tang, Guanyu Yao, Jun Yu

**Affiliations:** 1 School of Control Science and Engineering, Dalian University of Technology, Dalian 116024, China; 2 Key Lab of Liaoning for Integrated Circuits and Medical Electronic Systems, Dalian University of Technology, Dalian 116024, China; 3 School of Microelectronics, Dalian University of Technology, Dalian 116024, China; 4 Cancer Hospital of Dalian University of Technology, Liaoning Cancer Hospital & Institute, Shenyang 110801, China; 5 School of Biomedical Engineering, Dalian University of Technology, Dalian 116024, China

**Keywords:** DFT calculation, ethanol gas sensor, ethanol adsorption properties, PdO–SnO_2_ nanosheet

## Abstract

Pure SnO_2_ and 1 at.% PdO–SnO_2_ materials were prepared using a simple hydrothermal method. The micromorphology and element valence state of the material were characterized using XRD, SEM, TEM, and XPS methods. The SEM results showed that the prepared material had a two-dimensional nanosheet morphology, and the formation of PdO and SnO_2_ heterostructures was validated through TEM. Due to the influence of the heterojunction, in the XPS test, the energy spectrum peaks of Sn and O in PdO–SnO_2_ were shifted by 0.2 eV compared with SnO_2_. The PdO–SnO_2_ sensor showed improved ethanol sensing performance compared to the pure SnO_2_ sensor, since it benefited from the large specific surface area of the nanosheet structure, the modulation effect of the PdO–SnO_2_ heterojunction on resistance, and the catalyst effect of PdO on the adsorption of oxygen. A DFT calculation study of the ethanol adsorption characteristics of the PdO–SnO_2_ surface was conducted to provide a detailed explanation of the gas-sensing mechanism. PdO was found to improve the reducibility of ethanol, enhance the adsorption of ethanol’s methyl group, and increase the number of adsorption sites. A synergistic effect based on the continuous adsorption sites was also deduced.

## 1. Introduction

Ethanol is a common and valuable volatile organic solvent widely used in the chemical, manufacturing, food, environment, health care, and pharmaceutical industries [1]. Ethanol can quickly evaporate and diffuse in the air, forming ethanol gas. Ethanol gas irritates the human respiratory system and central nervous system. Long-term exposure to a certain amount of ethanol gas may cause symptoms such as breathing difficulties, drowsiness, dizziness, nausea, and headaches [2]. The threshold limit value (time-weighted average value) of the ethanol vapor proposed by the Occupational Safety and Health Administration (OSHA) and the American Conference of Governmental Industrial Hygienists (ACGIH) is 1000 ppm [3]. High concentrations of ethanol gas (3.3%~19%) may also cause severe fire and explosion accidents when exposed to fire. For drunk driving screening and alcohol dependence patients, the ethanol content in exhaled breath is the crucial detection indicator [4]. In China, the ethanol detection threshold for exhaled breath for determining a driving under the influence (DUI) charge is 0.086 mg/L (~42 ppm). Similarly, European countries have set similar threshold values for DUI [5]. Overall, ethanol gas sensors with diverse detection ranges have broad application prospects in air quality detection, explosive alarms, and exhaled breath analysis.

Semiconductor gas sensors utilizing SnO_2_ as the primary gas-sensitive material have been demonstrated to exhibit outstanding performance in detecting ethanol gas [6,7,8]. These SnO_2_ sensors offer numerous advantages, including high sensitivity, rapid response time, exceptional stability, long lifespan, and affordability, making them extensively utilized in various everyday applications. Researchers consistently strive to enhance gas-sensing capabilities by exploring novel synthesis approaches, modulating the materials’ morphology, and incorporating precise quantities of sensitizers such as metal oxides and noble metals. Additionally, in some work, density functional theory (DFT) calculations were employed to investigate the interaction between gases and materials at the atomic level [9,10], offering a fresh perspective on comprehending the gas adsorption process preceding the gas-sensitive oxidation reaction.

For instance, Li et al. manipulated the parameters of the hydrothermal method to synthesize SnO_2_ with diverse morphologies, demonstrating that the hollow-sphere-structured SnO_2_ displayed the lowest operational temperature, highest response, and superior selectivity. Through DFT calculations, an adsorption configuration of ethanol on the surface of SnO_2_, along with the electrical properties of SnO_2_, were elucidated, aiding in explaining the gas-sensing mechanism [11]. Li et al. prepared LaCoO_3_/SnO_2_ nanoflowers using a hydrothermal method, which showed a lower operating temperature and a higher response than pure SnO_2_. DFT calculations revealed stronger ethanol adsorption and a more extensive charge transfer on the LaCoO_3_/SnO_2_ surface than that on pure SnO_2_ and pure LaCoO_3_ surfaces, which can explain the mechanism of response enhancement [12]. Xiao et al. synthesized PdO-SnO_2_ hollow microcubes through precipitation annealing and chemical etching, which showed excellent sensitivity and selectivity to ethanol gas at 300 °C. The hollow microsphere structure provides a large specific surface area and adsorption sites, and the catalytic properties of PdO promote the chemical adsorption and dissociation of target gases [13]. Inderan et al. also synthesized Pd/PdO-SnO_2_ nanorods by hydrothermal method, exhibiting better gas-sensing performance than pure SnO_2_ and Ni-SnO_2_ nanorods [14]. It can be seen that the PdO-SnO_2_ material system shows excellent potential in ethanol detection because PdO has a high catalytic performance in ethanol [15]. Moreover, DFT calculations are also used to explore the sensing mechanism. Pan et al. studied the O_2_ adsorption on the PdO(101) surface by DFT method with both PBE and HSE exchange-correlation functional. Three adsorption configurations were obtained, which were in agreement with the results of the temperature programmed desorption experiment. The adsorption was highly related to the gap between the PdO d-band center and the lowest unoccupied molecular orbital (LUMO) of O_2_. This work proved that the PdO could facilitate the O_2_ adsorption process, which was a critical step for the oxidation reaction [16].

Despite some studies on the PdO-SnO_2_ ethanol gas sensors having been conducted, further exploration of the synthesis methods that produce PdO-SnO_2_ materials with diverse morphologies is still needed. In addition, DFT simulation calculations of ethanol adsorption behavior on the PdO-SnO_2_ surface have not yet been reported.

In this paper, 1 at.% PdO-SnO_2_ and SnO_2_ nanosheets were prepared using a simple hydrothermal reaction. Their structure and composition were analyzed by XRD, SEM, TEM, and XPS. The formation of PdO-SnO_2_ heterojunctions was demonstrated through TEM and XPS. Compared with pure SnO_2_ nanosheets, PdO-SnO_2_ nanosheets showed better ethanol-sensitive properties. The adsorption characteristics of ethanol on SnO_2_ and PdO(101)-SnO_2_(110) surfaces were studied using the DFT method. The enhancement effect of PdO was obtained, and a possible synergistic effect was proposed. The gas-sensing mechanism was analyzed through microstructures, catalytic effects, heterostructures, and DFT results.

## 2. Experimental Details

### 2.1. Preparation of the Gas-Sensing Materials

The SnO_2_ and PdO–SnO_2_ nanosheets were synthesized using a simple and efficient hydrothermal method. The experimental procedure followed a series of steps. Firstly, 100 mL of a white suspension containing 0.01 M SnCl_2_, 0.01 M CTAB, and 0.2 M NaOH was prepared. For the PdO–SnO_2_ material, 0.01 mmol PdCl_2_ (1 at.%) was added. The suspension was stirred for 60 min and transferred into a hydrothermal reaction vessel. The vessel was positioned in a drying oven and heated to 160 °C for 24 h to activate the hydrothermal reaction. After the reaction, the vessel was allowed to cool naturally to room temperature, and a suspension was obtained. The suspension was centrifuged to obtain the precipitate, which was then cleaned with water and ethanol. The centrifugation and cleaning process was repeated three times. The product was dried at 90 °C overnight and then annealed at 600 °C for two hours at a pressure of one atmosphere, which not only removed any organic matter and residual hydroxides but also promoted the crystallization of the oxides. Finally, the SnO_2_ and PdO–SnO_2_ nanosheet materials were obtained.

### 2.2. Characterization

The microstructure of the samples was investigated using a D/MAX 2400 X-ray powder diffractometer Rigaku, Japan (CuKα 1, λ = 0.154056 nm) to perform X-ray diffraction (XRD) analysis. The microscopic morphology of the materials was characterized using a ZEISS MERLIN field-emission scanning electron microscope (FESEM, Carl Zeiss, Oberkochen, Germany) and a Thermo Talos F200X transmission electron microscope (TEM, Thermo Fisher Scientific, Waltham, MA, USA). The composition and chemical states of the elements in the materials were analyzed using a Thermo Scientific Escalab 250XI X-ray photoelectron spectrometer (XPS, Thermo Fisher Scientific, Waltham, MA, USA). In the XPS test, the monochromator light source was Al Kα X-rays (1486.6 eV), the spot size was 400 μm, the base pressure was about 5 × 10^−9^ mbar, and a charge neutralizer was used. The charge correction was performed with the C 1s peak (284.8 eV) as a reference.

### 2.3. Fabrication and Measurement of Gas Sensors

The prepared material was fully ground and mixed with ethanol to form a slurry, which was then coated on a commercial ceramic sheet (3 mm × 3 mm × 0.25 mm) that was designed and fabricated for gas sensor applications. On the front of the ceramic sheet, a pair of Au electrodes was fabricated to test the resistance of the gas-sensitive material. On the back, a heating resistance wire was fabricated to heat the device. By applying different voltages to the heating resistance wire, the temperature of the ceramic sheet can be controlled from room temperature to 400 °C. The ceramic sheet coated with the gas-sensing materials was dried at 60 °C for 2 h and then annealed at 400 °C for 2 h. Finally, the gas sensor was prepared by mounting the ceramic sheet on a metal base in a suspended state through four leads.

The gas sensors’ properties were measured on a homemade gas-sensing test system described elsewhere [17]. The gas sources (Dalian Special Gas Products Co., Ltd., Dalian, China) included dry air and a standard gas containing the target gas at a specific concentration. Mass flow controllers were used to control the concentration of the target gas, which was introduced into a chamber containing the gas sensors. The resistances of the gas sensors were measured using a multimeter (Keithley DAQ6510, Tektronix, Beaverton, OR, USA). The resistances of a gas sensor in air and the target gas were marked *R*_a_ and *R*_g_, respectively. The response was defined as S = *R*_a_/*R*_g_. The response/recovery time was defined as the time corresponding to a 90% resistance change during the response/recovery stage [18,19].

### 2.4. Density Functional Theory Calculations

The DFT calculations were performed using the Dmol3 module in Materials Studio 2019 software. The Perdew–Burke–Ernzerhof (PBE) method within the framework of Generalized Gradient Approximation (GGA) was employed to describe the exchange-correlation energy. The DNP-4.4 basis set was chosen for the functional basis. This basis set was developed by Delley in 2006, which can give energy differences close to the DFT basis set limit [20,21]. Monireh et al. proved its high accuracy compared to the DND-4.4 and DNP-3.5 basis sets [22]. Meanwhile, employing the DNP-4.4 basis set in the Dmol3 module provides reasonable time and cost savings compared to the plane-wave method in the CASTEP module. A k-point mesh with a sampling size of 3 × 1 × 1 was utilized. During geometry optimization, the convergence criteria for energy, forces, and displacements were set to 1.0 × 10^−5^ Ha, 2 × 10^−3^ Ha·Å^−1^, and 5.0 × 10^−3^ Å, respectively.

The SnO_2_(110) and PdO(101)–SnO_2_(110) surfaces were constructed and are described in the Supplementary File. These surfaces were selected according to the TEM and XRD test results. The adsorption configurations of an ethanol molecule on the SnO_2_(110) and PdO(101)–SnO_2_(110) surfaces were computed. The adsorption energy was calculated using Equation (1) [23,24] where *E*_ads_ represents the adsorption energy, *E*_m+gas_ represents the energy of the system after the gas is adsorbed on the nanomaterial, *E*_m_ is the energy of the gas-sensing material, and *E*_gas_ is the energy of the gas molecule. A negative adsorption energy indicates a decrease in the system’s energy after gas adsorption, signifying a stable adsorption configuration where the gas molecule can spontaneously adsorb on the nanomaterial’s surface. Furthermore, the more negative the adsorption energy, the stronger the adsorption interaction is [25].

*E*_ads_ = *E*_m+gas_ − *E*_m_ − *E*_gas_ (1)

## 3. Results and Discussion

### 3.1. Characterization

**Figure 1:**
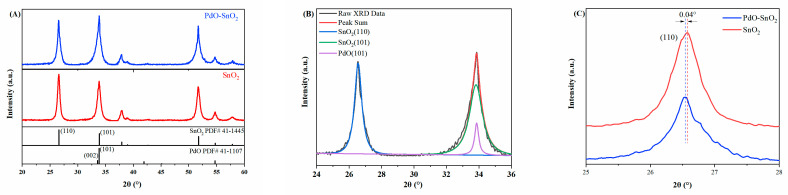
(**A**) XRD patterns of PdO–SnO_2_ and SnO_2_ materials. (**B**) Peak fitting results of the diffraction peaks of PdO–SnO_2_. (**C**) Magnified diffraction peaks of SnO_2_(110) crystal plane.

**Figure 2:**
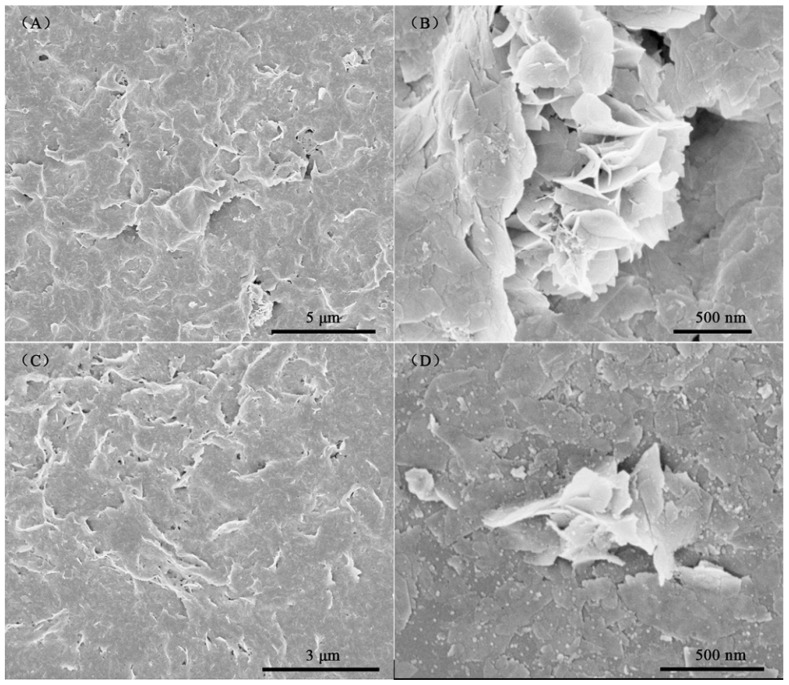
Low and high magnification SEM images of (**A**,**B**) SnO_2_ and (**C**,**D**) PdO–SnO_2_.

**Figure 3:**
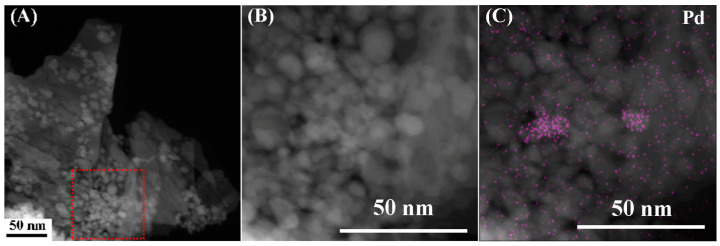

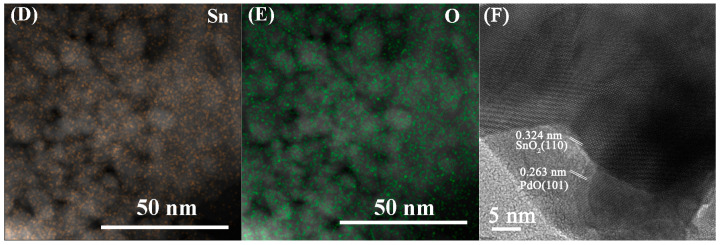
(**A**) The HAADF image of the PdO–SnO_2_ material, (**B**) the enlarged image of the red rectangle, and the EDS mapping images for (**C**) Pd, (**D**) Sn, and (**E**) O elements. (**F**) High-resolution TEM image of the PdO–SnO_2_ material.

The crystal structures of the prepared SnO_2_ and PdO–SnO_2_ materials were studied using XRD, and their XRD diffraction patterns are shown in Figure 1A. The diffraction peaks of the two materials can both be indexed to tetragonal SnO_2_. For SnO_2_, the highest diffraction peak at around 26.61° was derived from the SnO_2_(110) crystal plane, which is consistent with the standard pattern (JCPDS No. 41-1445). However, for PdO–SnO_2_, the highest diffraction peak appeared at around 33.86°, which is near the SnO_2_(101) peak and the PdO(101) peak (JCPDS No. 41-1107). It is unreasonable to infer that the highest diffraction peak comes only from the SnO_2_(101) crystal plane, because in previous studies, PdO did not have the function of regulating the growth orientation of SnO_2_ crystals [14,26]. In addition, the SEM results in Figure 2 do not show obvious changes in morphology or crystal orientation, while TEM observation in Figure 3F confirms the formation of PdO. Therefore, we infer that the PdO(101) diffraction peak exists and forms the highest diffraction peak after superposition with the SnO_2_(101) peak. Similar XRD results and explanations have also been reported by Amit [27]. The peak fitting results are shown in Figure 1B. Figure 1C shows the SnO_2_(110) diffraction peak patterns of the two materials. The SnO_2_(110) diffraction peak of PdO–SnO_2_ shifted by 0.04° to a low angle, indicating that the lattice of SnO_2_ increased after doping with PdO.

Microstructural characterization of the SnO_2_ and PdO–SnO_2_ materials was conducted using SEM. In Figure 2, a nanosheet structure is observed. These nanosheets were intertwined with each other. Some areas of the nanosheets assembled into a porous morphology, as shown in the high magnification image. These pores are beneficial in increasing the specific surface area of the material and promoting the diffusion and adsorption of gas within the material. The morphologies of the SnO_2_ and PdO–SnO_2_ materials were relatively similar, except that more small-sized nanoparticles were observed on the surface of PdO–SnO_2_.

The structural properties of the PdO–SnO_2_ material were analyzed more deeply using TEM. Figure 3A shows a low magnification HAADF (high angle annular dark field) image of the PdO–SnO_2_ material. Nanosheets loaded with or surrounded by nanoparticles were observed. The enlarged image of the red rectangle area is shown in Figure 3B. There were pores among the nanoparticles, facilitating the diffusion of gas molecules. Meanwhile, the nanosheets and nanoparticles with high specific areas provide abundant adsorption sites for gas molecules. The EDS mapping images in Figure 3B for Pd, Sn, and O are displayed in Figure 3C–E. Two nanoparticles exhibited a high content of Pd while other nanoparticles had a high content of Sn. O was evenly distributed in all nanoparticles. Therefore, two PdO nanoparticles and several SnO_2_ nanoparticles were found in this area. Figure 3F shows a high-resolution TEM image of the PdO–SnO_2_ material. The visible 0.263 nm and 0.324 nm lattice fringes were ascribed to the PdO(101) and the SnO_2_(110) crystal planes, respectively. The PdO nanoparticle is in contact with the edge of the SnO_2_ nanosheet, implying the formation of a PdO(101)–SnO_2_(110) heterojunction.

**Figure 4:**
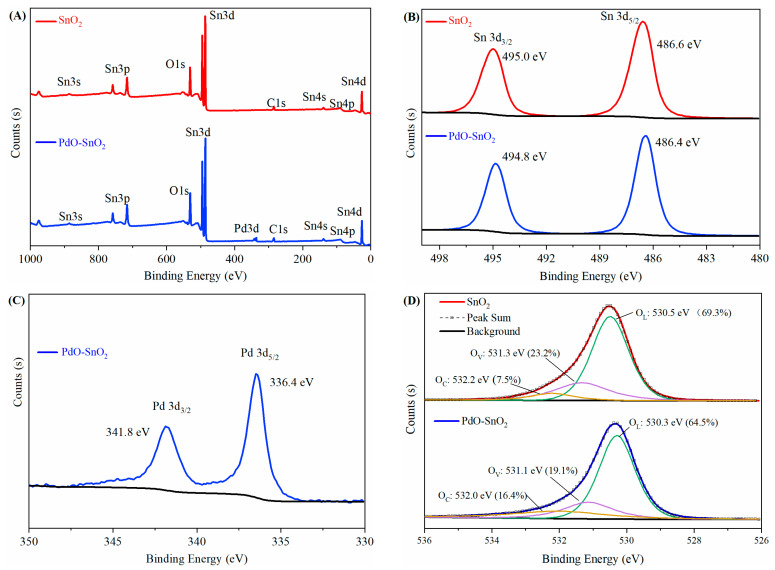
XPS spectra of the SnO_2_ and the PdO–SnO_2_ materials: (**A**) full-survey, (**B**) Sn 3d, (**C**) Pd 3d, and (**D**) O 1s. The black lines in the figures represent the signal background.

XPS was employed to characterize the elemental composition and chemical states of the materials. Figure 4A presents the full-survey spectra of the SnO_2_ and PdO–SnO_2_ samples. The results indicated the presence of Sn, O, and C in both samples and Pd in the PdO–SnO_2_ sample. Figure 4B displays the high-resolution XPS spectra for Sn 3d. Doublets with a peak separation of 8.4 eV were observed for both samples, which can be assigned to tetravalent Sn [28,29]. The binding energies of the PdO–SnO_2_ for Sn 3d were 0.2 eV lower than those of the SnO_2_, which can be attributed to the PdO doping [30]. Specifically, forming a heterojunction between PdO and SnO_2_ results in charge transfer, leading to changes in the density and energy of electrons in SnO_2_. Figure 4C shows the high-resolution XPS spectra of the PdO–SnO_2_ sample for Pd 3d. The Pd 3d_3/2_ and Pd 3d_5/2_ peaks were at 341.8 eV and 336.4 eV, indicating that Pd was divalent or there was PdO in the sample [13]. The Pd to Sn content measured using XPS was 1.43 at.%, close to the ratio of the raw materials. Figure 4D presents the high-resolution O 1s spectra. For the SnO_2_ sample, the O 1s peak can be deconvoluted (using Tougaard background) into three peaks located at 532.2 eV, 531.3 eV, and 530.5 eV, corresponding to chemisorbed O (O_C_), vacancy O (O_V_), and lattice O (O_L_), respectively [19,31,32]. The O_C_, O_V_, and O_L_ proportions were 7.5%, 23.2%, and 69.3%, respectively. For the PdO–SnO_2_ sample, the peaks for the O_C_, O_V_, and O_L_ were at 532.0 eV, 531.1 eV, and 530.3 eV, with proportions of 16.4%, 19.1%, and 64.5%, respectively. Similar to the Sn 3d spectra, the binding energies of the PdO–SnO_2_ material for O 1s also decreased due to the PdO doping. The PdO–SnO_2_ sample showed a higher O_C_ proportion than the SnO_2_ sample, which may have resulted from the PdO’s spillover effect. Meanwhile, The PdO–SnO_2_ sample had a lower O_V_ proportion. We inferred that this was also because of the PdO’s spillover effect. In the annealing process, the PdO could provide active oxygen species to the SnO_2_ surface, promoting surface oxidation and crystallization, thereby reducing oxygen vacancies. The O_V_ + O_C_ proportion is considered to be highly correlated with the responses of the gas sensors [31,32]. The O_V_, which represents the oxygen vacancies, can act as the oxygen adsorption sites. The O_C_ includes chemisorbed oxygen and other oxygen containing species such as OH^−^, H_2_O, etc. The chemisorbed oxygen can directly participate in the gas-sensing reaction. The amount of oxygen-containing species also reveals the material’s oxygen adsorption ability since these sites may transform into oxygen adsorption sites after desorption at a high working temperature. Moreover, Pd(OH)_2_ can react with reducing substances to generate CO_2_ and H_2_O [33]. Therefore, it is reasonable that the PdO–SnO_2_ gas sensor exhibited superior performance since its O_V_ + O_C_ proportion was 4.8% higher than that of the SnO_2_ sensor.

### 3.2. Gas-Sensing Properties

**Figure 5:**
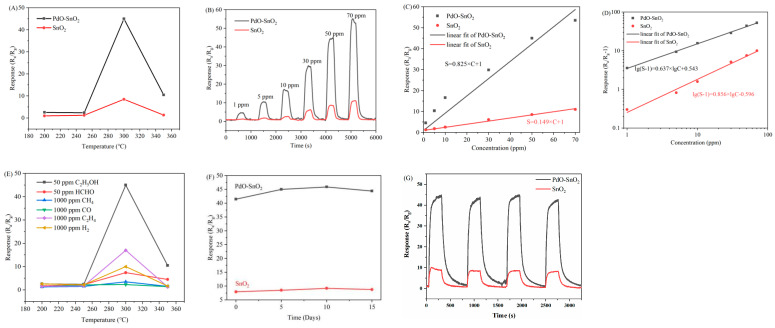
(**A**) Responses of PdO−SnO_2_ and SnO_2_ gas sensors to 50 ppm ethanol at different operating temperatures. (**B**) Responses to ethanol gas at different concentrations at an operating temperature of 300 °C. Responses as a function of ethanol concentration in (**C**) linear and (**D**) logarithmic coordinate systems. (**E**) Responses of the PdO−SnO_2_ gas sensor to ethanol and other interfering gases at different temperatures. (**F**) The long-term stability and (**G**) repeatability of the PdO−SnO_2_ and SnO_2_ gas sensors.

A series of gas-sensing tests were conducted to gain an in-depth understanding of the PdO–SnO_2_ and SnO_2_ nanosheet gas sensors. Temperature is an important parameter that affects the performance of gas sensors. In order to determine the appropriate temperature for sensor operation, the responses of the two sensors towards 50 ppm ethanol gas at 200–350 °C were tested. The results are shown in Figure 5A. As the temperature increased, the PdO–SnO_2_ and SnO_2_ gas sensors exhibited an “increase–decrease” trend. The trend can be explained as follows. The redox reaction at the materials’ surface, which determines the responses of the sensors, highly depends on the temperature. As the temperature increases from 200 °C to 300 °C, the adsorbed oxygen ions’ activity and the redox reaction are enhanced, increasing the response. When the temperature is above 300 °C, high temperature may suppress the adsorption of ethanol and oxygen, and carriers are also activated, which weakens the sensor’s sensitivity by decreasing *R*_a_ [17]. The highest response of PdO–SnO_2_ and SnO_2_ gas sensors were 45.0 and 8.5, respectively, when working at 300 °C, which was determined as the optimal operating temperature for the sensors. In addition, the PdO–SnO_2_ sensor, whose response was 5.3 times higher than that of the SnO_2_ sensor, exhibited a significant advantage. The reasons are analyzed in detail in Section 3.4.

Further investigation on the responses of PdO–SnO_2_ and SnO_2_ gas sensors to 1–70 ppm ethanol at 300 °C was carried out, and the result is depicted in Figure 5B. The responses of the two sensors had a positive correlation with the gas concentration. As the ethanol concentration rose from 1 ppm to 70 ppm, the PdO–SnO_2_ gas sensor’s response increased from 4.6 to 52.7, and the SnO_2_ gas sensor’s response grew from 1.3 to 11.1. In addition, the response time and recovery time of the two gas sensors to 50 ppm ethanol were also measured (Figure 5B). The response time and recovery time of the PdO–SnO_2_ gas sensor were 103 s and 149 s, respectively, and those of the SnO_2_ gas sensor were 120 s and 135 s.

The relationships between the sensors’ responses (S) and ethanol gas concentration (C) in the linear and logarithmic coordinate system are shown in Figure 5C and Figure 5D, respectively. Linear fits were performed in both coordinate systems. In the linear coordinate system, the relationships between the responses and the ethanol concentrations for the SnO_2_ and PdO–SnO_2_ gas sensors were fitted as S = 0.149 × C + 1 and S = 0.825 × C + 1, respectively, with R^2^ values of 0.997 and 0.973. Similarly, in the logarithmic coordinate system, the relationships between the responses and the ethanol concentrations for the SnO_2_ and PdO–SnO_2_ gas sensors were fitted as lg(S − 1) = 0.856 × lgC − 0.596 and lg(S − 1) = 0.637 × lgC + 0.543, respectively, with R^2^ values of 0.987 and 0.998. The closer to 1 the R^2^ value is, the smaller the fitting error is. Therefore, the SnO_2_ and PdO–SnO_2_ gas sensors showed good linear relationships between the responses and the ethanol concentrations in the linear and the logarithmic coordinate systems, respectively. Such a difference may be ascribed to the differences in the electronic characteristics and catalytic effects induced by PdO [15,17].

Selectivity is the ability of a gas sensor to detect target gases and eliminate the influence of interfering gases, which is very important for actual use. Figure 5E shows the responses of the PdO–SnO_2_ gas sensor to ethanol and other interfering gases, including formaldehyde, methane, ethylene, carbon monoxide, and hydrogen. The PdO–SnO_2_ gas sensor showed a response of 45.0 and 7.4 to 50 ppm ethanol and 50 ppm HCHO, respectively. The response ratio reached 6, indicating an excellent selectivity for ethanol and a poor selectivity for HCHO. The other interfering gases were tested at a concentration of 1000 ppm, and the responses were still far lower than that of 50 ppm ethanol, indicating an even poorer selectivity for these interfering gases. The results demonstrated the excellent selectivity of the PdO–SnO_2_ gas sensor to ethanol. This may be attributed to the catalyst effect of PdO and the strong adsorption of ethanol on the material’s surface [15,34].

Stability refers to the ability of the sensor to maintain sensitivity to the measured gas over a period of time. Figure 5F displays the responses of the two sensors to 50 ppm ethanol over 15 days. Small amplitude oscillations in the responses were observed, and no performance degradation or failure occurred, indicating that both sensors had remarkable stability. The repeatability of the sensors was also validated by cyclically measuring the responses to 50 ppm ethanol. As shown in Figure 5G, both the PdO–SnO_2_ and SnO_2_ sensors consistently exhibited stable gas-sensing responses across four cycles, demonstrating the excellent repeatability of the sensors.

A comparative analysis of the ethanol-sensing performances of other materials is depicted in Table 1. As a result, the PdO–SnO_2_ nanosheet sensor in this work achieved superior ethanol-sensing performance, particularly a strong response and a low limit of detection. These properties make this sensor a promising candidate for DUI detection and air quality monitoring.

### 3.3. DFT Calculation Results

The experiment results showed a significant increase in the response after doping PdO into SnO_2_. DFT calculations were also employed to investigate the adsorption characteristics of ethanol on the SnO_2_(110) and PdO(101)–SnO_2_(110) surfaces. The atom symbols used in the discussion are listed in Table 2.

**Figure 6:**
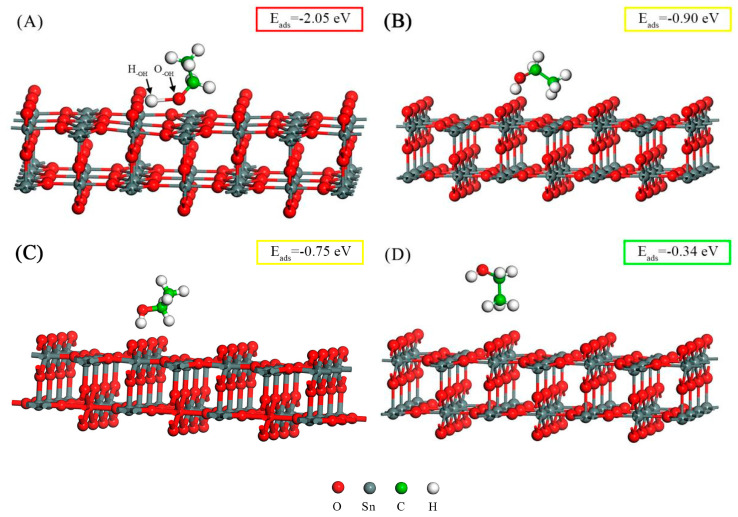
The adsorption configuration of a C_2_H_5_OH molecule on the SnO_2_(110) surface. (**A**) The −OH group is adsorbed on the Sn_5c_ and O_2c_(SnO_2_) atoms; (**B**) the H_−OH_ and the H_−CH3_ atoms are adsorbed on the O_2c_(SnO_2_) and Sn_5c_ atoms, respectively; (**C**) the H_−OH_ atom is adsorbed on the O_2c_(SnO_2_) atom; (**D**) the H_−CH3_ atoms are adsorbed on the O_2c_(SnO_2_) atom. The red, yellow, and green colors of the rectangle around *E*_ads_ indicate strong, medium, and weak adsorption strengths, respectively.

**Figure 7:**
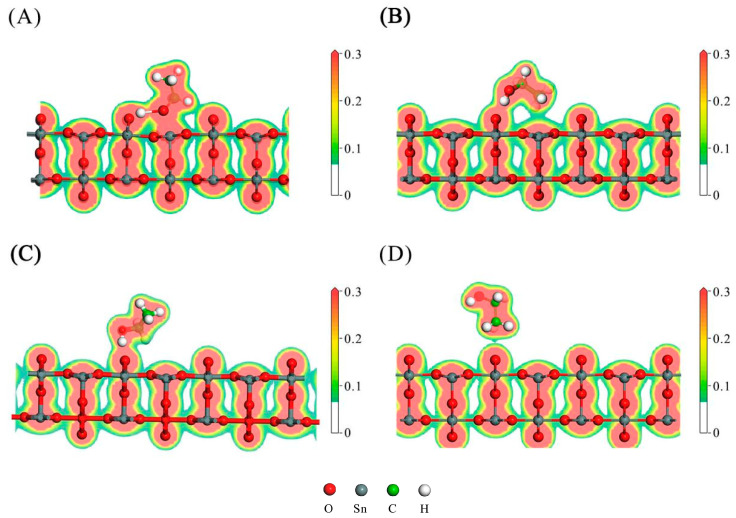
The charge density distribution of the adsorption configurations for an ethanol molecule adsorbed on the SnO_2_(110) surface, corresponding to Figure 6 (**A**–**D**).

The ethanol adsorption configurations on the SnO_2_(110) surface are illustrated in Figure 6, and the corresponding images of the charge density distribution are depicted in Figure 7. In this study, we first placed the ethanol molecule (with the −OH group pointing downward) above the Sn_5c_ and O_2c_(SnO_2_) atoms, and Figure 6A,C were obtained after geometry optimization. Then, we reversed the orientation of the ethanol molecule (with the -CH_3_ group pointing downward), and Figure 6B,D were obtained. The adsorption configurations in Figure 6 are sorted in descending order according to the absolute value of the adsorption energy. The optimal configuration had the most negative adsorption energy, indicating that its structure was the most stable. The charge density distribution images can be used to analyze the adsorption more intuitively. Adsorption occurs when the charge clouds of two atoms overlap, and a large charge density at the overlapping region indicates a strong adsorption.

Figure 6A corresponds to the optimal adsorption configuration with the most negative adsorption energy. In Figure 7A, it can be observed that the charge cloud of the H_-OH_ atom overlapped with that of the O_2c_(SnO_2_) atom on the SnO_2_(110) surface. Similarly, the charge cloud of the O_-OH_ atom overlapped with that of the Sn_5c_ atom. These overlapped clouds indicate the hybridization of the electronic orbitals between the ethanol molecules and the SnO_2_(110) surface. The substantial charge density (red color) between the adsorbed atoms reveals a robust adsorption procedure. The adsorption distance between the H_-OH_ atom and the O_2c_(SnO_2_) atom was 1.011 Å, and the adsorption distance between the O_-OH_ atom and the Sn_5c_ atom was 2.068 Å. The bond length between the O_-OH_ and H_-OH_ atoms significantly increased from 1.064 Å (in the free state) to 1.789 Å, suggesting that this adsorption configuration is conducive to the dissociation of the -OH group. Additionally, one H_-CH2_ atom also underwent charge exchange with the O_2c_(SnO_2_) atom on the right side. However, the charge density at the overlapping region was relatively low (green color), indicating a weaker adsorption effect. Therefore, the ethanol molecule primarily adsorbed onto the SnO_2_(110) surface through the -OH group. The adsorption energy for this configuration was −2.05 eV, and 0.151 e electrons were transferred from the ethanol molecular to the SnO_2_(110) surface.

The three additional ethanol adsorption configurations on the SnO_2_(110) surface are illustrated in Figure 6B–D, with their corresponding charge density distributions shown in Figure 7B–D. The adsorption energies for these three configurations were −0.90 eV, −0.75 eV, and −0.34 eV, respectively. The electrons transferred from the ethanol molecular to the SnO_2_(110) surface for the three configurations were 0.253 e, 0.227 e, and 0.146 e, respectively, which was positively related to the adsorption strength. However, the optimal adsorption configuration with the most robust interaction had few transferred charges (0.151 e) due to the adsorption of the O_-OH_ atom with a high affinity for electrons. The O_-OH_ atom received about 0.17 e more electrons than those in the other three configurations. Therefore, the total transferred charge was less.

To summarize the four configurations, the primary adsorption sites on the SnO_2_(110) surface were Sn_5c_ and O_2c_(SnO_2_). The adsorption strength was strongly related to the -OH group of the ethanol molecule: (a) a strong adsorption indicated that the H_-OH_ and O_-OH_ atoms were both adsorbed; (b) a medium adsorption indicated that the H_-OH_ atom was adsorbed, and the O_-OH_ atom was not; and (c) a weak adsorption indicated that the H_-OH_ and O_-OH_ atoms were not adsorbed, and only the H_-CH3_ atom was adsorbed.

**Figure 8:**
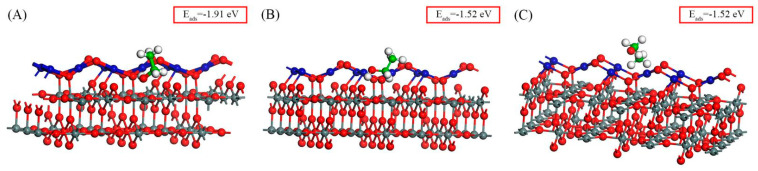

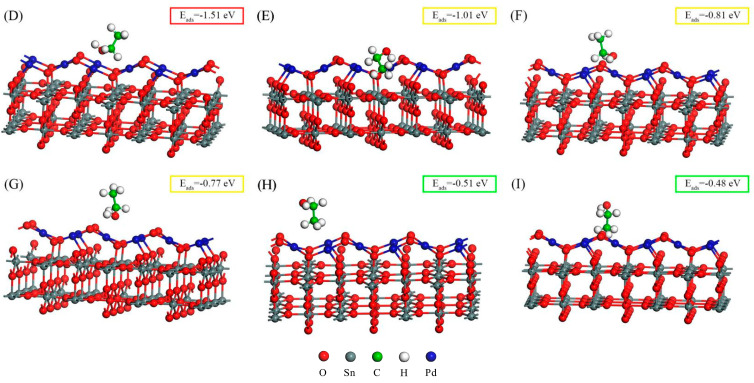
The adsorption configuration of a C_2_H_5_OH molecule on the PdO(101)−SnO_2_(110) surface. The −OH group is adsorbed on (**A**) Sn_5c_ and O_3c_ atoms; (**B**) Pd_3c_ and O_2c_(SnO_2_) atoms; (**C**) Pd_3c_ and O_3c_ atoms; and (**D**) Pd_3c_ and O_2c_ atoms. (**E**) The H_−OH_ and H_−CH2_ atoms are adsorbed on the O_2c_ and Pd_3c_ atoms, respectively. (**F**) The H_−OH_ atom is adsorbed on the O_2c_ atom. (**G**) The O_−OH_ and H_−OH_ atoms are adsorbed on the Pd_4c_ and O_2c_ atoms, respectively. (**H**) The H_−CH3_ atoms are adsorbed on the Pd_3c_ and Pd_4c_ atoms. (**I**) The H_−CH3_ atoms are adsorbed on the O_2c_ and O_2c_(SnO_2_) atoms. The red, yellow, and green colors of the rectangle around *E*_ads_ indicate strong, medium, and weak adsorption strengths, respectively.

**Figure 9:**
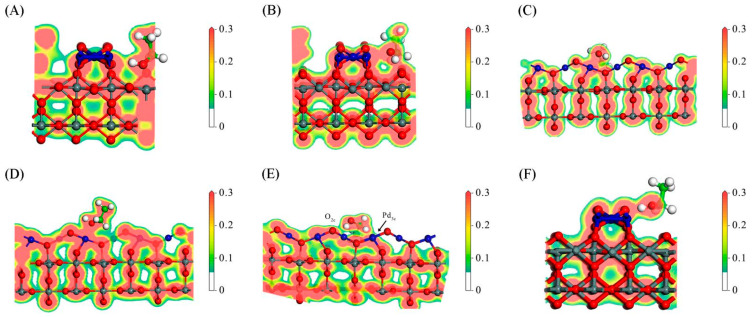

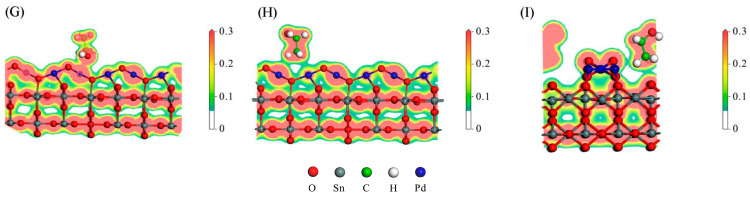
The charge density distribution of the adsorption configurations for an ethanol molecule adsorbed on the PdO(101)–SnO_2_(110) surface, corresponding to Figure 8 (**A**–**I**).

On the PdO(101)–SnO_2_(110) surface, nine adsorption configurations were obtained through DFT calculations, as depicted in Figure 8. In the calculations, we first placed the ethanol molecule (with the -OH group pointing downward) above the Pd_3c_ and Pd_4c_ atoms, and obtained Figure 8D and 8G, respectively. When the ethanol molecule was placed above the oxygen atoms, an adsorption configuration similar to Figure 8D could be obtained due to the strong interaction between the O_-OH_ and Pd_3c_ atoms. Then, we placed the ethanol molecule with the -OH group pointing downward and approached the Pd_3c_, O_3c_, and O_2c_ atoms from the side, and obtained Figure 8B, 8A, and 8F, respectively. Afterward, we placed the ethanol molecule (with the -CH_3_ group pointing downward) above the Pd atoms (each H atom above one Pd atom) and obtained Figure 8H. Then, we pointed the -CH_3_ group downward and approached the O_2c_ atom from the side to obtain Figure 8I. When the -CH_3_ group of the ethanol molecule was pointed downward and to the side of the O_3c_ atom, the -OH group would be adsorbed because of the low position of the O_3c_ atom, and Figure 8C,E were obtained depending on the adsorption of the -OH group. The adsorption configurations in Figure 8 are sorted in descending order according to the absolute value of the adsorption energy. We believe these typical configurations cover the majority of the adsorption situations and can provide us with sufficient information to analyze the adsorption characteristics. Figure 9 shows the charge density distribution of the configurations in Figure 8, allowing us to analyze the adsorption more intuitively and accurately.

Figure 8A shows the optimal adsorption configuration with the most negative adsorption energy. In Figure 9A, the O_-OH_ atom was strongly adsorbed on the Sn_5c_ atom due to the large charge density between the two atoms. Meanwhile, the H_-OH_ atom was adsorbed on the O_3c_ atom, and their charge density was smaller, indicating a weaker interaction. In this configuration, the ethanol molecule was adsorbed on both the PdO and SnO_2_ surfaces. Also, 0.266 e electrons were transferred from ethanol to the PdO–SnO_2_ surface.

The adsorption energies for the configurations in Figure 8B–D were all approximately −1.50 eV, exhibiting a strong adsorption. Their distinctive feature was that the O_-OH_ and H_-OH_ atoms participated in adsorption simultaneously. The O_-OH_ atoms were adsorbed on the Pd_3c_ atom, while the H_-OH_ atom was adsorbed on one O atom. From the charge density distribution images in Figure 9B–D, it is evident that there was a significant overlap between the charge clouds of O_-OH_ and Pd_3c_, exhibiting a strong adsorption interaction. Meanwhile, the adsorption of H_-OH_ with the O atom exhibited a weaker interaction due to a lower density of the overlapped charge cloud. Hence, the Pd_3c_ atom acted as a crucial adsorption site on the PdO surface. However, compared with the optimal adsorption configuration, the ethanol adsorption capability of Pd_3c_ seemed weaker than that of Sn_5c_. The electrons transferred from ethanol to the PdO–SnO_2_ surface were 0.252 e, 0.247 e, and 0.229 e in the three configurations.

In Figure 8G, the O_-OH_ atom and the H_-OH_ atom were adsorbed on the Pd_4c_ and the O_2c_ atoms, respectively. Even though both the O_-OH_ atom and the H_-OH_ atom participate in adsorption, the adsorption energy was only −0.77 eV, much smaller than those in Figure 9B–D. This reduced adsorption capability may be because the Pd_4c_ atom had already been coordinated with four surrounding lattice oxygen atoms. This configuration showed a moderate adsorption strength. The electrons transferred from ethanol to the PdO–SnO_2_ surface in this configuration were 0.180 e.

The configurations in Figure 8E,F had adsorption energies of −1.01 eV and −0.81 eV, respectively, which are moderate adsorption strengths. In both configurations, only the H_-OH_ atom was adsorbed on the O_2c_ atom, while the O_-OH_ atom was not adsorbed. Notably, the adsorption was stronger in the configuration in Figure 8E, which was attributed to the additional adsorption interaction between the H_-CH2_ atom and the Pd_3c_ atom. The electrons transferred from ethanol to the PdO–SnO_2_ surface were 0.286 e and 0.229 e in the two configurations.

The adsorption energies for the configurations in Figure 8H,I were around −0.5 eV, indicating weak adsorption strengths. In these configurations, the H_-CH3_ atoms participated in the adsorption, and the -OH group did not. The results suggest that the H_-CH3_ atom was far less activated than the O_-OH_ and H_-OH_ atoms. The electrons transferred from ethanol to the PdO–SnO_2_ surface were 0.186 e and 0.157 e, respectively.

Similar to the SnO_2_(110) surface, the -OH group also had a significant impact on the adsorption on the PdO(101)–SnO_2_(110) surface. The adsorption characteristics on the SnO_2_(110) and PdO(101)–SnO_2_(110) surfaces are summarized in Table 3. The conditions for different adsorption strengths are listed in Table 4.

After ethanol is adsorbed, electrons are transferred from ethanol molecules to the PdO(101)–SnO_2_(110) surface, so ethanol exhibits a reducing property. However, there is no positive correlation between transferred electrons and adsorption strength because the O_-OH_ atom gains electrons if adsorbed. Considering the strong affinity of the O_-OH_ atom for electrons, we divided the adsorption configurations on the PdO(101)–SnO_2_(110) surface into two categories based on whether the O_-OH_ atom was adsorbed. In Table 3, the corresponding number of transferred electrons is displayed by underlines based on when the O_-OH_ atom is adsorbed. In each category, the transferred electrons and the adsorption strength exhibited a positive correlation.

By comparing the adsorption characteristics on the two surfaces, the following conclusions were drawn:

- According to the adsorption energy in Table 3, the ethanol adsorption strength was stronger on the Sn_5c_ atom (*E*_ads_ ≈ −2 eV) than on the Pd_3c_ atom (*E*_ads_ ≈ −1.5 eV). However, comparing the optimal adsorption configurations on two surfaces, the transferred electrons were increased by 0.115 e on the PdO(101)–SnO_2_(110) surface, indicating a higher reducing property of ethanol with the assistance of PdO. Furthermore, for the weak adsorption configurations, the adsorption energy was more negative on the PdO(101)–SnO_2_(110) surface, revealing the enhancement of the adsorption of the H_-CH3_ atoms by PdO.
- On the PdO(101) surface, it was found that the adsorption sites were continuously distributed since every atom of PdO can act as an adsorption site. On the SnO_2_(110) surface, the Sn_5c_ and O_2c_(SnO_2_) atoms acted as the adsorption sites with a relatively sparse distribution. Therefore, PdO can facilitate ethanol adsorption through numerous adsorption sites.

### 3.4. Gas-Sensing Mechanism

It is generally believed that the oxidation reaction between ethanol and oxygen ions adsorbed on the surface causes a change in SnO_2_-based gas sensors’ resistance. In the air, oxygen can be adsorbed on the surface of SnO_2_ and capture electrons from SnO_2_, forming oxygen ions such as O_2_^−^, O^−^, and O^2−^ [45]. A depletion layer at the surface of SnO_2_ is simultaneously formed, which increases the sensor’s resistance. When ethanol arrives, it firstly adsorbs on the SnO_2_’s surface, and then reacts with the pre-adsorbed oxygen ions, producing CO_2_ and H_2_O, as shown in Equations (2)–(4) [7,46]. Through this reaction, the electrons captured by the oxygen ions escape and flow back into the SnO_2_ conduction band, causing a decrease in the thickness of the depletion layer and the sensor’s resistance [47].

C_2_H_5_OH + 3O_2_^−^ = 2CO_2_ + 3H_2_O + 3e^−^ (2)

C_2_H_5_OH + 6O^−^ = 2CO_2_ + 3H_2_O + 6e^−^ (3)

C_2_H_5_OH + 6O^2−^ = 2CO_2_ + 3H_2_O + 12e^−^ (4)

**Figure 10:**
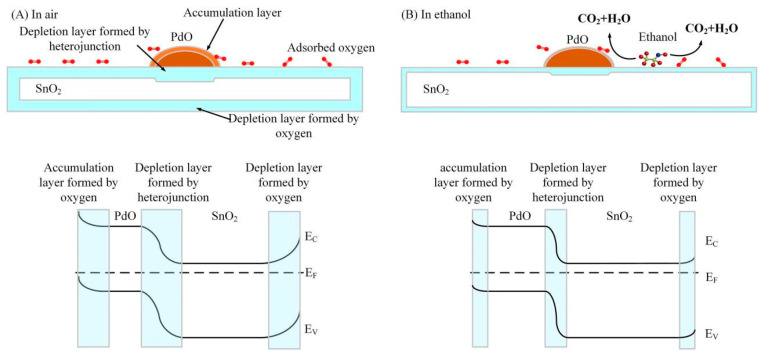
The schematic diagram of the gas-sensing mechanism and energy band diagram of the PdO–SnO_2_ sensor in (**A**) air and (**B**) ethanol gas.

A heterojunction is formed between the p-type PdO (*E*_g_ = 3.84 eV) and n-type SnO_2_ (*E*_g_ = 3.6 eV) [48,49]. Since the work function of PdO (7.9 eV) is higher than that of SnO_2_ (4.5 eV) [30], electrons flow from SnO_2_ into PdO and form a depletion layer at the PdO–SnO_2_ interface. In the air, oxygen can be adsorbed on p-type PdO, capturing electrons and forming an accumulation layer on the PdO with a high hole concentration. The increase in hole concentration means that the Fermi level of the PdO is reduced, so more electrons flow from the SnO_2_ into PdO, increasing the width of the depletion layer at the PdO–SnO_2_ interface. When ethanol arrives, it also consumes the adsorbed oxygen ions on the PdO surface. Electrons escape from oxygen and enter the PdO valence band to recombine with holes, causing the hole concentration to decrease and the Fermi level to increase. Therefore, the electrons flow back into SnO_2_, causing the thickness of the depletion layer and the resistance to decrease. The schematic diagrams of the gas-sensing mechanism and the energy band diagram on the SnO_2_ surface and at the PdO–SnO_2_ interface are shown in Figure 10.

In the gas-sensing test, the PdO–SnO_2_ gas sensor showed excellent gas-sensing performance, which is due to the following four reasons:

1. The PdO–SnO_2_ material has a two-dimensional nanosheet structure, which gives it a large specific surface area and many adsorption sites [37,50,51].
2. PdO has a catalyst effect on ethanol oxidation [15,17]. Specifically, PdO can enhance the amount of oxygen adsorbed on the SnO_2_ surface through the spillover effect [52,53,54], thereby enhancing the gas-sensing reaction on the SnO_2_ surface. In addition, PdO can also lower the chemical reaction barrier [55].
3. The heterostructure can enhance the gas-sensing performance [56,57]. Due to the high catalytic performance of PdO, the gas-sensing reaction can cause significant changes in PdO’s hole concentration and Fermi level, which can be reflected in the thickness change of the depletion layer and the resistance at the heterojunction.
4. According to the DFT calculation results, PdO has the following enhancement effects: First, it enhances the charge transfer amount after adsorption and increases the reducing performance of ethanol. Second, it enhances the adsorption strength of H_-CH3_ atoms on the material surface. Third, PdO has a large number of continuous adsorption sites, increasing the adsorption probability of ethanol. Furthermore, we propose a possible synergistic effect. Because the adsorption sites are continuous on the PdO surface, the ethanol molecule can move to adjacent adsorption sites through a small amount of energy exchange with the environment. Therefore, ethanol molecules exhibit a certain degree of mobility on the PdO surface. Through this mobility, the ethanol molecule may move to the interface between PdO and SnO_2_, react with the spillover oxygen, and thereby change the resistance of SnO_2_. This inferred process can be viewed as a synergistic effect.

## 4. Conclusions

Two-dimensional SnO_2_ and 1 at.% PdO–SnO_2_ nanosheet materials were synthesized using a facile hydrothermal method. The microscopic morphology and elemental states of the materials were characterized using XRD, SEM, TEM, and XPS. Two-dimensional nanosheets were stacked to form a gas-sensitive thin film, with some regions exhibiting a porous morphology due to the assembly of nanosheets. The TEM observations revealed the heterogeneous junction structure of PdO–SnO_2_. XPS characterization confirmed the presence of Sn^4+^, O^2−^, and Pd^2+^ in the PdO–SnO_2_ material and spectral peak shifts due to the PdO–SnO_2_ heterojunctions. The gas-sensing tests demonstrated that the PdO–SnO_2_ gas sensors exhibited excellent gas-sensing properties at 300 °C, significantly outperforming the pure SnO_2_ material. This outstanding gas-sensing performance can be attributed to the large surface area of the nanosheet structure, the modulation effect of PdO–SnO_2_ heterojunctions, and the catalyst effect of PdO. The DFT calculations revealed the adsorption characteristics of ethanol on the SnO_2_(110) and PdO(101)–SnO_2_(110) surfaces. The enhancement effect of PdO was analyzed, and a possible synergistic effect that improved the gas-sensing reaction was proposed.

## Figures and Tables

**Table 1:** Ethanol-sensing performances of different gas-sensing materials.

Material	Temp. (°C)	Con. (ppm)	Res. (*R*_a_/*R*_g_)	LOD (ppm)	Ref.
ZnO–In_2_O_3_	350	10	5	1	[35]
Pb–In_2_O_3_	250	100	32.57	5	[36]
In_2_O_3_–ZnO	225	100	52	0.2	[37]
Zn_2_SnO_4_–RGO	275	100	38	5	[38]
α–Fe_2_O_3_	300	100	37.57	5	[39]
MoS_2_–ZnO	220	500	12.08	-	[40]
HoFeO_3_	280	100	33	5	[41]
SnO_2_–CuO	320	100	8	-	[42]
SnO_2_	350	100	27.13	2.94	[43]
TiO_2_–SnO_2_	260	50	7.54	1	[44]
PdO–SnO_2_	300	1	4.6	1	This work
		10	16.7		
		70	52.7		

Temp.: temperature. Con.: concentration. Res.: response. LOD: limit of detection. Ref.: reference.

**Table 2:** The atom symbols and their meanings.

Atom Symbol	Meaning
O_−OH_	The O atom of the −OH group in the ethanol molecule
H_−OH_	The H atom of the −OH group in the ethanol molecule
H_−CH2_	The H atom of the −CH_2_ group in the ethanol molecule
H_−CH3_	The H atom of the −CH_3_ group in the ethanol molecule
Sn_5c_	The Sn atom coordinated with five O atoms on the SnO_2_(110) surface
O_2c_(SnO_2_)	The O atom coordinated with two Sn atoms on the SnO_2_(110) surface
Pd_3c_	The Pd atom coordinated with three O atoms on the PdO(101)–SnO_2_(110) surface
Pd_4c_	The Pd atom coordinated with four O atoms on the PdO(101)–SnO_2_(110) surface
O_2c_	The O atom coordinated with two Pd atoms on the PdO(101)–SnO_2_(110) surface
O_3c_	The O atom coordinated with two Pd atoms and one Sn atom on the PdO(101)–SnO_2_(110) surface

**Table 3:** The adsorption properties of SnO_2_(110) and PdO(101)–SnO_2_(110) surfaces.

Surface	Adsorption Configuration	Adsorption Energy (eV)	Transferred Electrons (e)	Adsorbed Atom Pair	Adsorption Strength
SnO_2_(110)	Figure 6A	−2.05	0.151 *	O_-OH_-Sn_5c_; H_-OH_-O_2c_(SnO_2_); H_-CH2_-O_2c_(SnO_2_)	strong
	Figure 6B	−0.90	0.253	H_-OH_-O_2c_(SnO_2_); H_-CH3_-Sn_5c_	medium
	Figure 6C	−0.75	0.227	H_-OH_-O_2c_(SnO_2_)	medium
	Figure 6D	−0.34	0.146	H_-CH3_-O_2c_(SnO_2_)	weak
PdO(101)– SnO_2_(110)	Figure 8A	−1.91	0.266	O_-OH_-Sn_5c_; H_-OH_-O_3c_	strong
	Figure 8B	−1.52	0.252	O_-OH_-Pd_3c_; H_-OH_-O_2c_ (SnO_2_)	strong
	Figure 8C	−1.52	0.247	O_-OH_-Pd_3c_; H_-OH_-O_3c_	strong
	Figure 8D	−1.51	0.229	O_-OH_-Pd_3c_; H_-OH_-O_2c_	strong
	Figure 8E	−1.01	0.286	H_-OH_-O_2c_; H_-CH2_-Pd_3c_	medium
	Figure 8F	−0.81	0.229	H_-OH_-O_2c_	medium
	Figure 8G	−0.77	0.18	O_-OH_-Pd_4c_; H_-OH_-O_2c_	medium
	Figure 8H	−0.51	0.186	H_-CH3_-Pd_3c_; H_-CH3_-Pd_4c_	weak
	Figure 8I	−0.48	0.157	H_-CH3_-O_2c_; H_-CH3_-O_2c_(SnO_2_)	weak

* The underlines indicate the O−OH atom is adsorbed in the configurations.

**Table 4:** Adsorption conditions that determine the adsorption strength.

Adsorption Strength	Adsorption Conditions		
	O_-OH_	H_-OH_	H_-CH3_
strong	adsorbed on Sn_5c_ or Pd_3c_	adsorbed	-
medium	adsorbed on Pd_4c_ or not adsorbed	adsorbed	-
weak	not adsorbed	not adsorbed	adsorbed

## Data Availability

The data will be made available upon request.

## Supplementary Materials

The following supporting information can be downloaded at: https://www.mdpi.com/article/10.3390/s24154970/s1, Figure S1: (A) 3D view, (B) left view, and top view of the 3 × 3 × 2 supercell of the SnO_2_(110) surface; Figure S2: (A) 3D view, (B) left view, and top view of the PdO(101)–SnO_2_(110) heterostructure.
