# Supplementary material for: Experimental and Density Functional Theory Simulation Research on PdO–SnO2 Nanosheet Ethanol Gas Sensors
Source: Sensors (Basel). 2024 Jul 31;24(15):4970. doi: 10.3390/s24154970 (PMC11314670; doi:10.3390/s24154970)
Supplement: Supplementary file 1 [file sensors-24-04970-s001.zip › sensors-3133438-supplementary.pdf]

Experimental and DFT simulation research on SnO_2 and PdO-SnO_2 nanosheet ethanol gas sensors

Wu Hao, Zhang Jianwei, Zhu Huichao, Li Xiaogan, Liu Hongxu, Tang Zhenan, Yao Guanyu, Yu Jun

Figure S1 shows the supercell structure of the 3×3×2 SnO_2(110) crystal plane, which contains 6 layers of atoms. A vacuum layer with a thickness of 15Å is set above the crystal plane. The basis vectors of the SnO_2(110) crystal plane are u=3.2837Å, v=6.7796Å, and the angle between the basis vectors is θ=90°. There are two types of Sn atoms with five coordination and six coordination on the surface, which are recorded as Sn_{5c} and Sn_{6c} respectively; there are also two-coordinated bridge oxygen and three-coordinated planar oxygen, which are recorded as $O_{2c}(SnO_2)$ and $O_{3c}(SnO_2)$ respectively.

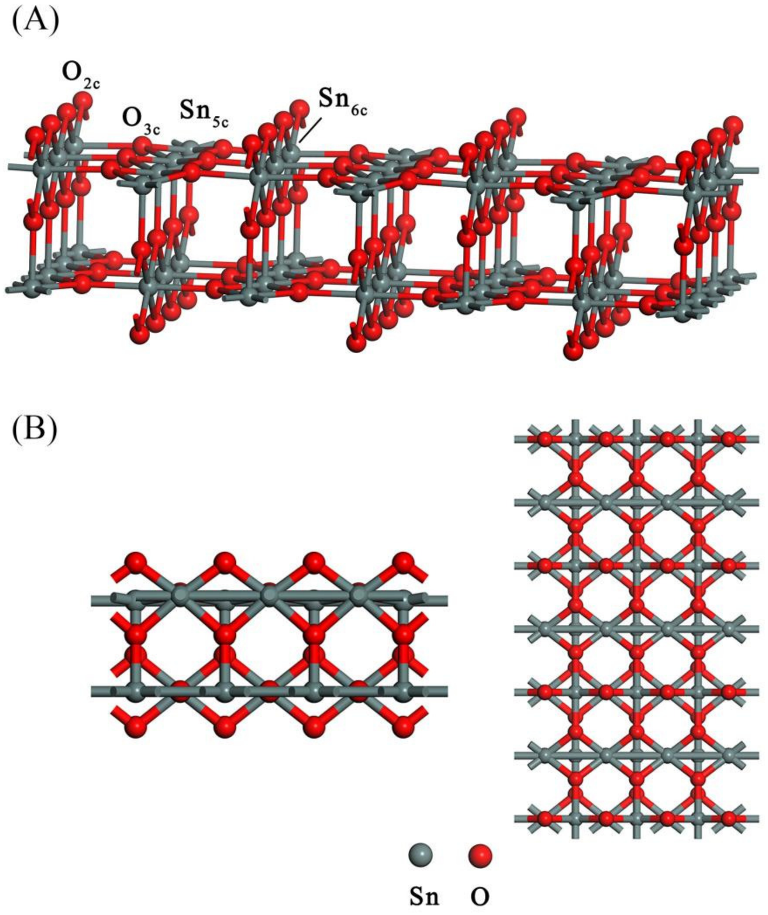

Figure S1. (**A**) 3D view, (**B**) left view, and top view of the 3×3×2 supercell of SnO_2(110) surface

Figure S2 shows the model of PdO-SnO_2 heterojunction. The basis vectors of the PdO(101) crystal plane are u=3.1827Å, v=6.1861Å, and the basis vector angle is θ=90°. The basis vector lengths of PdO(101) and SnO_2(110) crystal planes are similar, and the angle between the basis vectors are both 90°, so they can be constructed into a heterostructure. The 1×3×1 supercell of the PdO(101) crystal plane was placed above

the 3×3×2 supercell of the SnO_2(110) crystal plane. On the surface of PdO(101), there are Pd atoms that are three-coordinated or four-coordinated with the surrounding O atoms, recorded as Pd_{3c} and Pd_{4c} respectively. There are also O atoms that are two-coordinated or three-coordinated with the surrounding atoms, recorded as O_{2c} and O_{3c} respectively. Since the basis vector length of the SnO_2(110) crystal plane is larger than that of the PdO(101) crystal plane, the atomic distance between the bottom of the PdO(101) crystal plane and the SnO_2(110) crystal plane is larger, and outwards expands, while the atoms at the top of the PdO(101) crystal plane shrink inward. For example, the distance between adjacent O_{3c} atoms is 3.164Å, while the distance between adjacent O_{2c} atoms is 2.401Å.

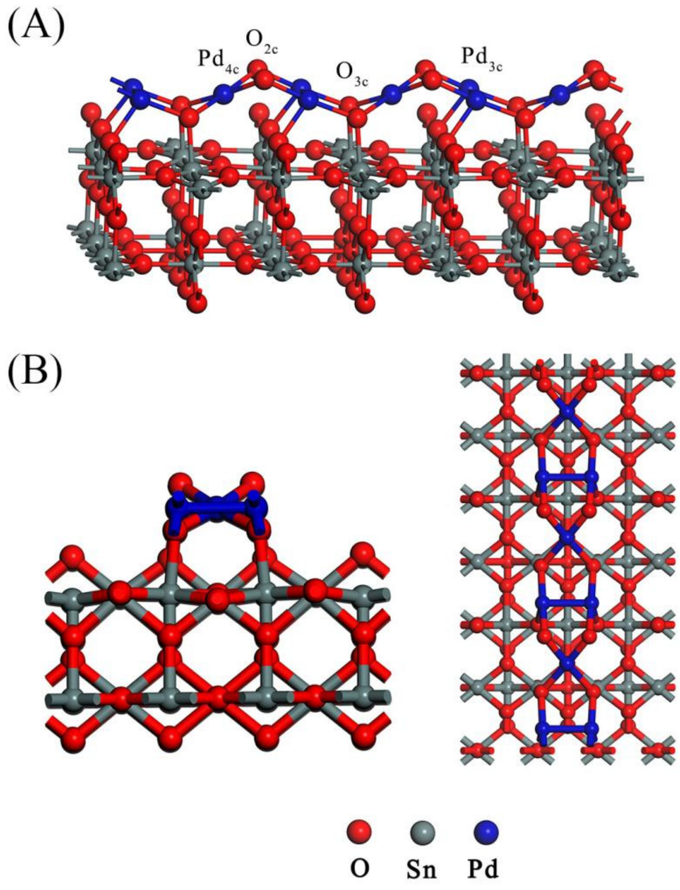

Figure S2. (**A**) 3D view, (**B**) left view, and top view of the PdO(101)-SnO_2(110) heterostructure
